# Carcinosarcoma is an aggressive subtype of bladder cancer: A population‐based study

**DOI:** 10.1002/cam4.4611

**Published:** 2022-02-18

**Authors:** Lin Liu, Jinglan Zhu, Yun Tian

**Affiliations:** ^1^ Zhaoqing Medical College Zhaoqing China; ^2^ The Central Hospital of Shaoyang Shaoyang China; ^3^ Affiliated Cancer Hospital & Institute of Guangzhou Medical University Guangzhou China

**Keywords:** Bladder carcinosarcoma, prognosis, SEER database

## Abstract

**Background:**

Case reports of bladder carcinosarcoma (BCS) indicate high rates of recurrence and metastasis and poor prognosis. However, the differences in clinicopathologic characteristics and prognosis between BCS and conventional bladder cancer histologies (transitional cell carcinoma [TCC], squamous cell carcinoma [SCC] and adenocarcinoma [AC]) have not been fully clarified in a large study. Therefore, we conducted a large population‐based study to further investigate these differences.

**Patients and methods:**

Information on patients with BCS and conventional bladder cancer (TCC, SCC or AC) was extracted from the Surveillance, Epidemiology, and End Results database. Categorical variables were compared using Pearson's chi‐squared test or Fisher's exact test. Survival analysis was carried out using the Kaplan–Meier method, and differences in survival were assessed using the log‐rank test. Propensity score matching analysis was conducted to calibrate the differences between the baseline characteristics, after which Cox regression analysis was applied to calculate the hazard ratios and 95% confidence intervals of BCS compared to other subtypes. Subgroup analysis and related interaction were tested to evaluate the consistency and heterogeneity of results.

**Results:**

We enrolled 152 patients with BCS and 180,196 patients with TCC, SCC or AC. Our results showed that BCS was associated with poor differentiation, advanced stage and an unfavourable overall survival and cancer‐specific survival. BCS had a worse prognosis than TCC and AC, but no statistically significant difference in survival was noted between BCS and SCC.

**Conclusions:**

BCS is a more aggressive bladder cancer than TCC and AC but is comparable to SCC. These findings broaden our understanding of BCS and may be helpful in clinical practice.

## Graphical abstract

Bladder carcinosarcoma was associated with poor differentiation, presented at an advanced stage, and was related with an unfavourable overall survival and cancer‐specific survival. Additionally, carcinosarcoma had a worse prognosis than transitional cell carcinoma and adenocarcinoma, but no statistically significant difference in survival was noted between carcinosarcoma and squamous cell carcinoma in bladder cancer.

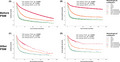

## 1 BACKGROUND

Bladder cancer is the most common carcinoma of the urinary system, and it is characterised by high morbidity and mortality.^1^ An estimated 573,278 new bladder cancer cases were diagnosed in 2020 worldwide, and there were 212,536 associated deaths.^2^ More than 90% of bladder cancers are transitional cell carcinomas (TCCs), which is the most predominant histological subtype.^3^ The remaining 10% of bladder tumours include squamous cell carcinoma (SCC), adenocarcinoma (AC), carcinosarcoma (CS) and so on.^4^

Bladder CS (BCS) is defined by the World Health Organization (WHO) as a biphasic tumour with both malignant epithelial and mesenchymal components.^5^ Published case reports indicate that BCS has high rates of recurrence and metastasis, and patients present with poor differentiation and advanced stage, leading to poor prognosis.^6^, ^7^, ^8^, ^9^ In addition, BCS is thought to have outcomes similar to those of bladder TCC.^10^ The sample sizes of previous studies are too small to provide conclusive results.^6^, ^7^, ^8^, ^9^, ^10^ Therefore, a large population‐based study with the intent to compare BCS and conventional bladder cancer (TCC, SCC or AC) is warranted. In this study, we reviewed the Surveillance, Epidemiology, and End Results (SEER) database to compare clinicopathological characteristics and outcomes of patients with BCS and conventional bladder cancer.

## 2 PATIENTS AND METHODS

### 2.1 Data resource

Our research data were obtained from the SEER database (https://seer.cancer.gov/, accession number: 15095‐Nov2020). The SEER Program of the National Cancer Institute is an authoritative source of information on cancer incidence and survival in the United States.^11^ SEER currently collects and publishes cancer data from population‐based cancer registries covering approximately 47.9% of the United States population.^11^

### 2.2 Patient selection

Eligible patients were diagnosed with urinary bladder cancer between 2000 and 2018. Patients were selected only if the histology was CS, TCC, SCC or AC. We used the following WHO International Classification of Diseases for Oncology, third histology codes to identify the patients: CS, 8980–8981; TCC, 8120, 8122 and 8130–8131; SCC, 8070–8072, 8074–8076 and 8083; and AC, 8140, 8255, 8260–8263, 8310, 8323, 8480–8481, 8490 and 8503.^12^ The diagnosis was microscopically confirmed using surgery or biopsy specimens. Patients identified based on death certificate or autopsy only and those with more than one primary tumour or unavailable follow‐up data were excluded.

### 2.3 Survival outcome

Our main endpoints were overall survival (OS) and cancer‐specific survival (CSS). OS was calculated from the initial diagnosis to death from any cause or the last follow‐up. CSS was calculated from the initial diagnosis to death from cancer. The relevant survival data (survival months, vital status and cause of death) were extracted from the database.

### 2.4 Potential covariate

Covariates of interest included the age at diagnosis (<60 years or ≥60 years), sex (male or female), race (White, Black or others), marital status (married, unmarried or unknown), year of diagnosis (2000–2008 or 2009–2018), grade (grade I, well‐ differentiated; grade II, moderately differentiated; grade III, poorly differentiated; or grade IV, undifferentiated or unknown), SEER stage (localised, regional, distant or unknown), surgery (yes or no/unknown), radiation (yes or no/unknown), chemotherapy (yes or no/unknown) and histological subtype (CS, TCC, SCC or AC) based on the SEER program guidelines (https://seer.cancer.gov).

### 2.5 Statistical analysis

We downloaded the original information from the SEER*Stat software (version 8.3.9.2). Categorical variables are shown as numbers (frequencies and percentages) and were compared using Pearson's chi‐squared test or Fisher's exact test. Survival curves were generated using the Kaplan–Meier method, and differences between groups were compared using the log‐rank test. A propensity score matching (PSM) analysis at a 1:3 ratio was conducted to calibrate the differences between baseline characteristics. The Cox proportional hazard model was applied to calculate hazard ratios (HRs) and 95% confidence intervals (CIs) using BCS patients as the reference. Subgroup analysis was performed using the univariate regression analysis. All covariates were subdivided except for surgery status and histological subtype. The surgery status was not analysed in the subgroup analysis because the sample size was small. Interactions between histological subtype and other potential covariates were tested for both OS and CSS. We used SPSS 11.0 and R 3.6.3 for all statistical analyses.

## 3 RESULTS

### 3.1 Patient selection

**FIGURE 1:**
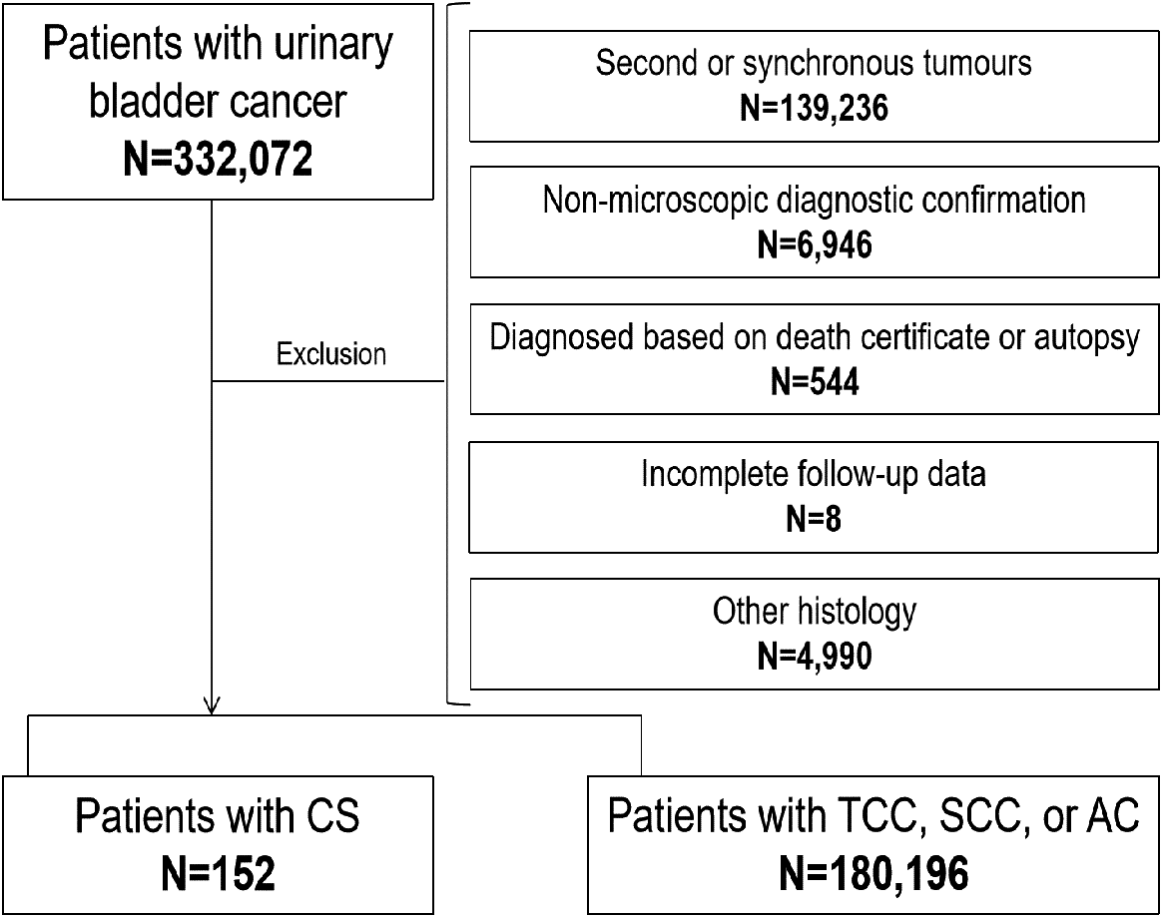
The patient selection process. AC, adenocarcinoma; CS, carcinosarcoma; SCC, squamous cell carcinoma; TCC, transitional cell carcinoma

In total, 332,072 patients with urinary bladder cancer were extracted from the SEER 18 registries from 2000 to 2018. After excluding ineligible patients, 152 patients with BCS and 180,196 patients with TCC, SCC or AC were enrolled in the final study. Figure 1 illustrates the patient selection process in a flow diagram.

### 3.2 Baseline characteristic

Tables 1 and 2 summarise the baseline characteristics of all identified patients. Of the BCS patients, 130 (85.5%) were aged 60 years or older, and the median age was 74 years (range: 33–96 years). Of these, most patients were male (63.8%), White (86.2%), married (53.9%) and diagnosed between 2000 and 2008 (51.3%). The most common treatment method was cancer‐directed surgery (96.1%); fewer patients received radiation (17.1%) or chemotherapy (26.3%).

**TABLE 1:** Basic characteristics of all patients

Covariates	Histological subtypes			
	CS	TCC	SCC	AC
Total	152 (100%)	175,780 (100%)	2490 (100%)	1926 (100%)
*Age, years*				
<60	22 (14.5%)	36,720 (20.9%)	574 (23.1%)	758 (39.4%)
≥60	130 (85.5%)	139,060 (79.1%)	1916 (76.9%)	1168 (60.6%)
*Sex*				
Male	97 (63.8%)	130,746 (74.4%)	1129 (45.3%)	1154 (59.9%)
Female	55 (36.2%)	45,034 (25.6%)	1361 (54.7%)	772 (40.1%)
*Race*				
White	131 (86.2%)	155,370 (88.4%)	2071 (83.2%)	1472 (76.4%)
Black	17 (11.2%)	9463 (5.4%)	309 (12.4%)	296 (15.4%)
Others	4 (2.6%)	10,947 (6.2%)	110 (4.4%)	158 (8.2%)
*Marital status*				
Married	82 (53.9%)	102,008 (58.0%)	992 (39.8%)	1472 (76.4%)
Unmarried	63 (41.4%)	59,845 (34.0%)	1383 (55.5%)	296 (15.4%)
Unknown	7 (4.6%)	13,927 (7.9%)	115 (4.6%)	158 (8.2%)
*Year of diagnosis*				
2000–2008	78 (51.3%)	73,998 (42.1%)	1182 (47.5%)	885(46.0%)
2009–2018	74 (48.7%)	101,782 (57.9%)	1308 (52.5%)	1041 (54.0%)
*Grade*				
I	1 (0.7%)	20,003 (11.4%)	197 (7.9%)	128 (6.6%)
II	1 (0.7%)	45,331 (25.8%)	743(29.8%)	433(22.5%)
III	43 (28.3%)	29,098 (16.6%)	788 (31.6%)	602 (31.3%)
IV	64 (42.1%)	47,188 (26.8%)	273 (11.0%)	241 (12.5%)
Unknown	43(28.3%)	34,160 (19.4%)	489 (19.6%)	522 (27.1%)
*SEER stage*				
Localised	33 (21.7%)	109,328 (62.2%)	328 (13.2%)	337 (17.5%)
Regional	82 (53.9%)	25,634 (14.6%)	1225 (49.2%)	819 (42.5%)
Distant	19 (12.5%)	5455 (3.1%)	489 (19.6%)	369 (19.2%)
Unknown	18 (11.8%)	35,363 (20.1%)	448 (18.0%)	401 (20.8%)
*Surgery*				
No/Unknown	6 (3.9%)	9842 (5.6%)	368 (14.8%)	247 (12.8%)
Yes	146 (96.1%)	165,938 (94.4%)	2122 (85.2%)	1679 (87.2%)
*Radiation*				
No/Unknown	126 (82.9%)	167,454 (95.3%)	2053 (82.4%)	1676 (87.0%)
Yes	26 (17.1%)	8317 (4.7%)	437 (17.6%)	250 (13.0%)
*Chemotherapy*				
No/Unknown	112 (73.7%)	140,014 (79.7%)	1884 (75.7%)	1350 (70.1%)
Yes	40 (26.3%)	35,766 (20.3%)	606 (24.3%)	576 (29.9%)

Abbreviations: AC, adenocarcinoma; CS, carcinosarcoma; SCC, squamous cell carcinoma; TCC, transitional cell carcinoma.

**TABLE 2:** Comparison between patients with BCS and those with other bladder cancer histological subtypes (TCC, SCC and AC) before and after PSM

Covariates	*p*‐value					
	CS and TCC		CS and SCC		CS and AC	
	Before PSM	After PSM	Before PSM	After PSM	Before PSM	After PSM
Age	0.052	0.786	**0.014**	0.119	<**0.001**	0.163
Sex	**0.003**	0.428	<**0.001**	0.293	0.344	0.664
Race	**0.002**	**0.030**	0.499	0.093	**0.011**	0.800
Marital status	0.082	0.829	**0.002**	0.094	0.630	0.947
Year of diagnosis	**0.021**	<**0.001**	0.357	0.110	0.202	0.888
Grade	<**0.001**	<**0.001**	<**0.001**	<**0.001**	<**0.001**	<**0.001**
SEER stage	<**0.001**	<**0.001**	**0.002**	0.115	**0.002**	0.182
Surgery	0.376	0.242	<**0.001**	0.456	**0.001**	0.456
Radiation	<**0.001**	0.287	0.889	0.588	0.149	0.900
Chemotherapy	0.068	0.261	0.582	0.753	0.351	0.409

Abbreviations: AC, adenocarcinoma; CS, carcinosarcoma; PSM, propensity score matching; SCC, squamous cell carcinoma; TCC, transitional cell carcinoma.

Bold indicates significance level alpha (0.05).

Advanced stage (regional or distant) was more prevalent in patients with CS than in those with TCC (66.4% vs. 17.7%, *p* < 0.001) and AC (66.4% vs. 61.7%, *p* < 0.05), whereas it was less prevalent in patients with CS than in those with SCC (66.4% vs. 68.8%, *p* < 0.05). More than half of the CS patients had high grade (III–IV) tumours, whereas less than half of the TCC, SCC and AC patients did (43.4%, 42.6% and 43.8%, respectively; *p* < 0.001, *p* < 0.001 and *p* < 0.001, respectively). Patients with CS were more likely to receive surgical treatment than patients with SCC (96.1% vs. 85.2%, *p* < 0.001) or AC (96.1% vs. 87.2%, *p* = 0.001). More CS patients than TCC patients underwent radiation therapy (17.1% vs. 4.7%, *p* < 0.001). More details of the characteristics are presented in Tables 1 and 2.

### 3.3 Survival analysis

The median follow‐up in the entire cohort was 46 months (range: 0–227 months). One hundred and twenty‐six (82.9%) patients with CS died during the follow‐up period. Of these, 101 (66.4%) deaths were caused by CS. The CS group had a shorter median OS time than the TCC (8 months vs. 114 months) and AC (8 months vs. 28 months) groups. There was no significant difference in median OS time between the CS and SCC groups (8 months vs. 6 months). Similar results were observed in the CSS analysis.

**FIGURE 2:**
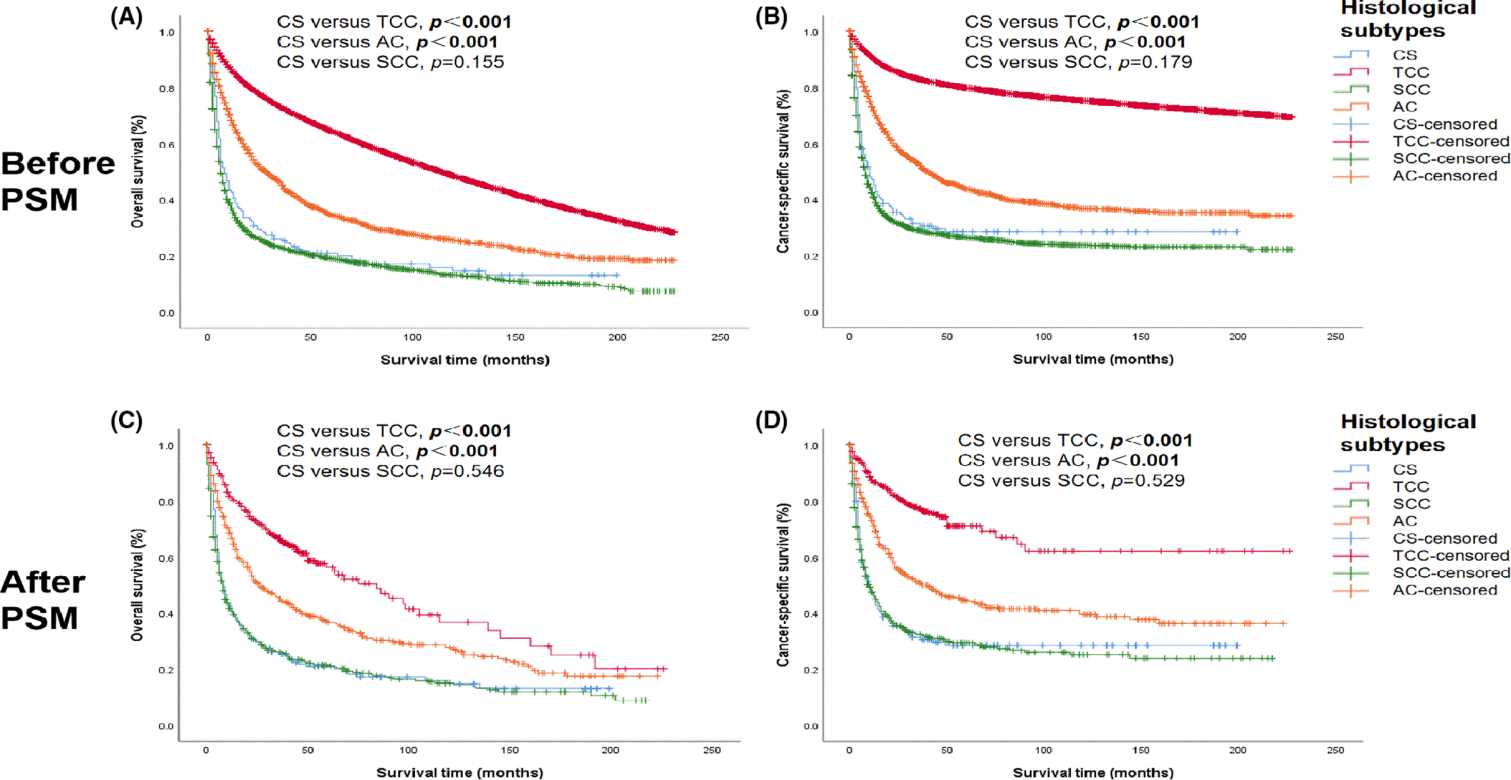
Survival analysis of patients according to the histological subtype of bladder cancer before and after PSM. A and B, overall survival (OS) and cancer‐specific survival (CSS) of all patients before PSM; C and D, OS and CSS after PSM. AC, adenocarcinoma; CS, carcinosarcoma; PSM, propensity score matching; SCC, squamous cell carcinoma; TCC, transitional cell carcinoma

The Kaplan–Meier plot indicated worse OS in patients with CS than in patients with TCC (*p* < 0.001) and AC (*p* < 0.001). There was no significant difference in OS between the CS and SCC groups (*p* = 0.155). Similar outcomes were observed in the CSS analysis. The survival probability of all patients is indicated in Figure 2.

To explore the prognostic differences between groups, we performed a 1:3 (CS: TCC/SCC/AC) PSM analysis to minimise the unevenness of the baseline characteristics, and most factors were matched. No significant difference was observed in the survival outcomes before and after PSM analysis. Detailed information is illustrated in Figure 2.

### 3.4 Cox proportional hazard model

**FIGURE 3:**
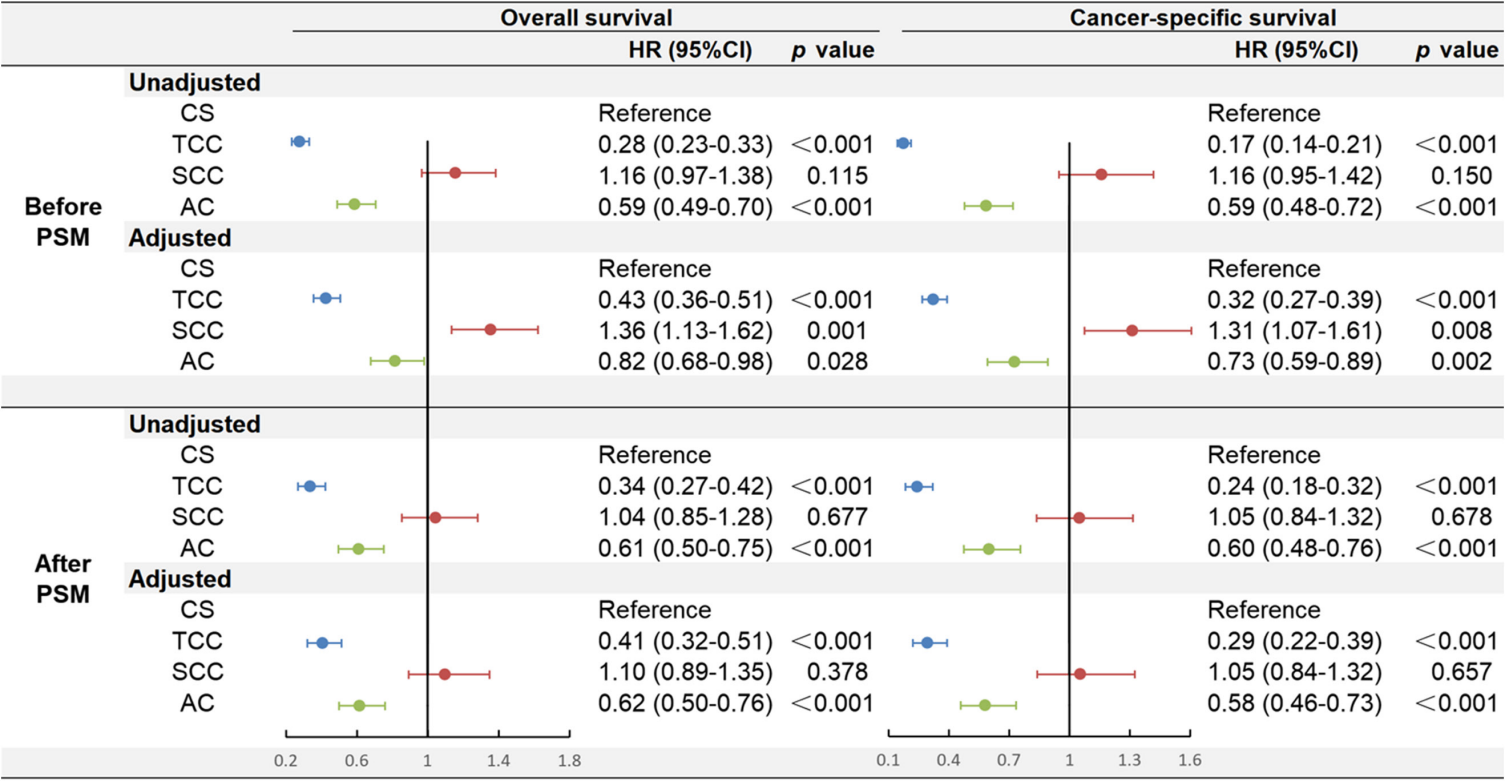
The Cox proportional hazards model for overall survival (OS) and cancer‐specific survival (CSS) before and after PSM. AC, adenocarcinoma; CI, confidence interval; CS, carcinosarcoma; HR, hazard ratio; PSM, propensity score matching; SCC, squamous cell carcinoma; TCC, transitional cell carcinoma

Cox regression and PSM analyses were performed to further identify the prognostic differences between the study groups. The two models (unadjusted and adjusted) before and after PSM analysis are presented in Figure 3. After PSM analysis and adjustments for all potential covariates except for histological subtype, multivariate Cox analysis also revealed that CS patients had worse OS than TCC (HR = 0.41, 95% CI: 0.32–0.51, *p* < 0.001) and AC (HR = 0.62, 95% CI: 0.50–0.76, *p* < 0.001) patients. We also observed an inferior CSS for CS patients compared with TCC (HR = 0.29, 95% CI: 0.22–0.39, *p* < 0.001) and AC (HR = 0.58, 95% CI: 0.46–0.73, *p* < 0.001) patients. Patients with CS and SCC had similar OS (HR = 1.10, 95% CI: 0.89–1.35, *p* = 0.378) and CSS (HR = 1.05, 95% CI: 0.84–1.32, *p* = 0.657).

### 3.5 Subgroup analysis

**FIGURE 4:**
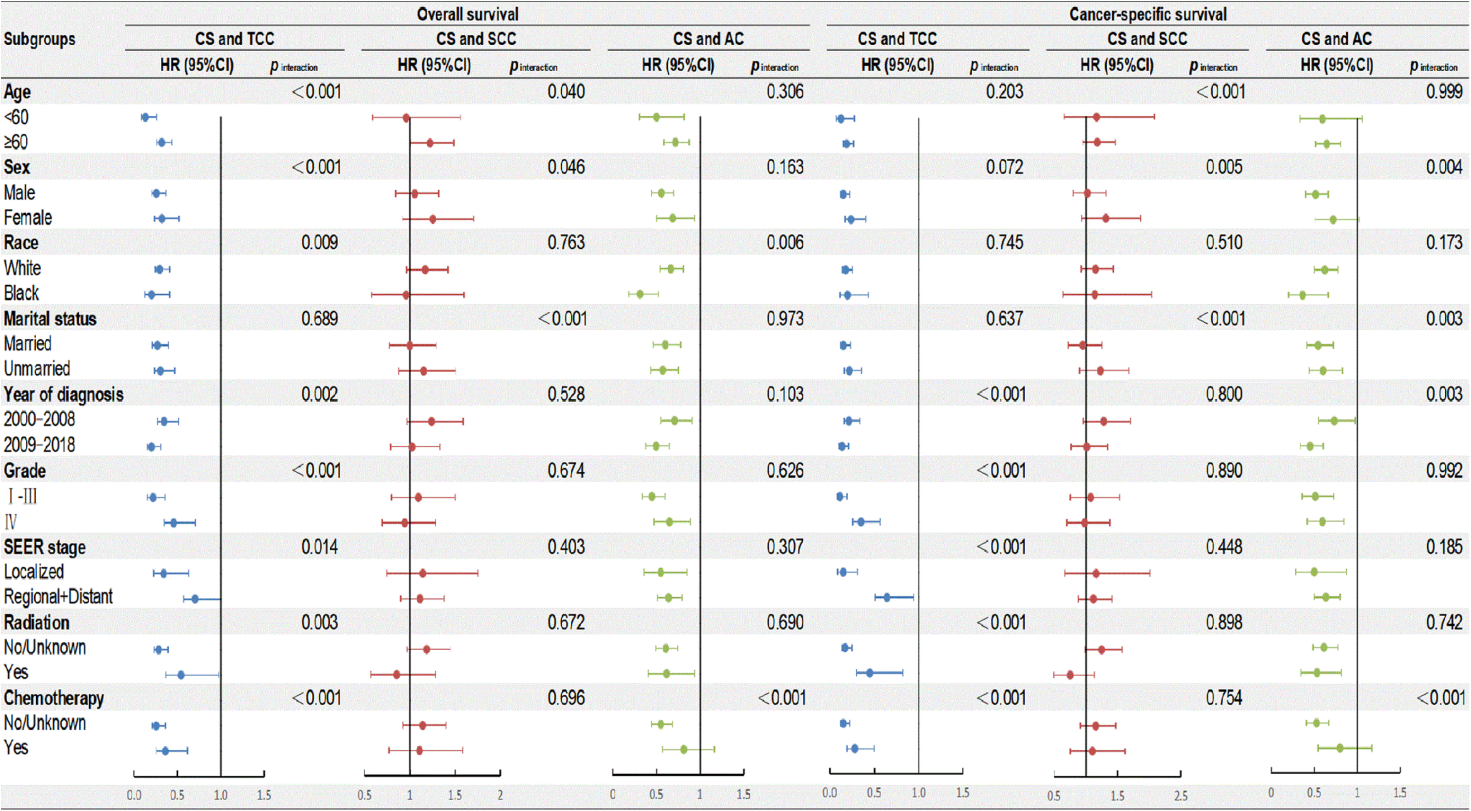
Subgroup analysis for interaction between histological subtype and potential covariates for both overall survival (OS) and cancer‐specific survival (CSS). HRs were calculated for comparison of CS and other histological subtypes (TCC, SCC and AC). AC, adenocarcinoma; CI, confidence interval; CS, carcinosarcoma; HR, hazard ratio; SCC, squamous cell carcinoma; TCC, transitional cell carcinoma

To verify the consistency of the relationships between histological subtype and survival outcomes, subgroup analyses were conducted. The OS and CSS of patients in the subgroups are shown as forest plots in Figure 4. We further performed interaction tests to evaluate the heterogenic effects of the histological subtype in different subgroups. As illustrated in Figure 4, statistically significant differences were observed in the interaction tests. Despite some heterogeneity, CS patients had worse OS and CSS than TCC patients in all analyses. CS patients also had worse OS and CSS than AC patients in most of the analyses. However, CS and SCC patients generally had a similar OS and CSS.

## 4 DISCUSSION

The goal of this study was to conduct a comparison between BCS and conventional bladder cancer (TCC, SCC or AC) rather than merely investigating the features of BCS.^12^ BCS is a rare tumour, and there is limited information on its characteristics and prognosis. Most of the available literature is case reports and literature reviews with small numbers of cases.^6^, ^7^, ^8^, ^9^ To the best of our knowledge, no prospective study or clinical trial on BCS has been published, and only two retrospective studies published in English included a large sample size.^12^, ^13^, ^14^ One identified 221 BCS patients in the SEER 17 registries diagnosed between 1973 and 2004; the other enrolled 3007 patients with genitourinary sarcomas diagnosed from 1973 to 2015 from the SEER 18 registries.^12^, ^13^ In the present study, we enrolled 152 BCS patients and 180,196 patients with TCC, SCC or AC diagnosed between 2000 and 2018 from the SEER 18 registries. Nazemi et al. reported that BCS had a median OS of 9 months and exhibited the shortest survival among genitourinary sarcomas arising in the bladder.^13^ Wang et al. collected 132 BCS patients and revealed that the median CSS was 14 months.^12^ These findings support our result that BCS is a highly aggressive subtype with worse prognosis. Nazemi et al. also evaluated the clinicopathological features of patients with BCS and found that patients tended to be older men who presented with advanced stage and higher grade.^13^ These results are similar to our findings.^6^, ^7^, ^8^, ^9^, ^14^, ^15^, ^16^, ^17^, ^18^, ^19^ Further, Nazemi et al. showed that most patients (95.9%) underwent cancer‐directed surgery, whereas only a small proportion of patients received radiation (16.7%).^13^ We observed similar characteristics in our cohort.

A previous study concluded that CS did not have a worse prognosis than TCC.^10^ However, we found that CS was a more aggressive tumour and was associated with a poor outcome than TCC. Survival analysis also indicated poorer prognosis for CS than for AC; no statistically significant difference in prognosis was noted between CS and SCC. Similar results were found in the Cox regression analysis, PSM analysis and subgroup analysis.

Some inherent limitations of this study should be acknowledged. First, the SEER database is an imperfect program in which there is incomplete information for many patients. The treatment strategies for such patients are not clear. We categorised these patients into the ‘no treatment’ group. Additionally, it was not possible to determine the pathological grade or SEER stage in 10%–30% of patients. This information might have made a difference in the research conclusions. Second, subgroup analysis revealed mild inconsistency, which might be partially caused by the small number of BCS cases. In addition, heterogeneity was observed in the interaction tests. An interaction between histological subtype and a covariate indicates that the covariate may be a stratifying factor for prognosis, but it could be an illusion or an artefact. Given the small number of cases, we cannot make any definite conclusions. Therefore, further large prospective studies with long‐term follow‐up are warranted to confirm our findings. Last, the enrolled patients were mostly White, and only a fraction of the patients were Black or other races. In view of these limitations, our current conclusions may not be generalisable to other populations.

The present study also has the following merits. First, we included 152 patients with BCS as the study group and 180,196 patients with conventional bladder cancer (TCC, SCC or AC) as the control group. The adequate number of cases made it possible to perform more standard analyses. Second, through series comparisons between the study and control groups, we further explored the characteristics and prognosis of BCS. Third, BCS is well known for its poor prognosis. We were curious whether BCS patients had worse survival than those with conventional bladder cancer (TCC, SCC or AC). Our findings revealed an inferior survival for BCS patients compared with TCC and AC patients, whereas similar survivals were observed between BCS and SCC patients. Last, to verify the consistency of survival outcomes, this study conducted a number of analyses (survival analysis, PSM analysis, Cox regression analysis and subgroup analysis). These methods are particularly important in promoting the power of statistical tests.

## 5 CONCLUSION

BCS occurred more commonly in older men, presented at an advanced stage, and was associated with poor differentiation and unfavourable OS and CSS. In addition, BCS was more aggressive than TCC and AC but comparable to SCC in aggressiveness. These findings broaden our understanding of BCS and may be helpful in clinical practice.

## CONFLICT OF INTEREST

The authors declare that there is no conflict of interest.

## AUTHOR CONTRIBUTIONS

All authors contributed to the design, data collection and analysis of the study and drafted the manuscript. All authors have read and approved the final manuscript.

## ETHICS STATEMENT

Since all SEER data are anonymous and publicly accessible, ethics board review and informed consent from the patient were not required.

## Data Availability

Publicly available databases were analysed in this study. These data can be found in the SEER database (https://seer.cancer.gov/).

## References

[1] Lenis AT , Lec PM , Chamie K . Bladdercancer: a review. JAMA. 2020;324:1980‐1991. 33201207 10.1001/jama.2020.17598

[2] Sung H , Ferlay J , Siegel RL , et al. Global cancer statistics 2020: GLOBOCAN estimates of incidence and mortality worldwide for 36 cancers in 185 countries. CA Cancer J Clin. 2021;71:209‐249. 33538338 10.3322/caac.21660

[3] Ismaili N , Arifi S , Flechon A , et al. Small cell cancer of the bladder: pathology, diagnosis, treatment and prognosis. Bull Cancer. 2009;96:E30‐E44. 19457759 10.1684/bdc.2009.0883

[4] Ploeg M , Aben KK , Hulsbergen‐van de Kaa CA , et al. Clinical epidemiology of nonurothelial bladder cancer: analysis of The Netherlands Cancer Registry. J Urol. 2010;183:915‐920. 20083267 10.1016/j.juro.2009.11.018

[5] Wick MR , Swanson PE . Carcinosarcomas: current perspectives and an historical review of nosological concepts. Semin Diagn Pathol. 1993;10:118‐127. 8367621

[6] Basibuyuk I , Topaktaş R , Elbir F . Bladder carcinosarcoma: a case report with review of the literature. Arch Ital Urol Androl. 2017;89:240‐242. 28969410 10.4081/aiua.2017.3.240

[7] Althubiany HH , Hasan RM , Alzahrani SA , Bahdilh S . Case report of a rareurinarybladdertumor variant (carcinosarcoma). Urol Ann. 2020;12:190‐192. 32565662 10.4103/UA.UA_76_19 PMC7292428

[8] Atılgan D , Gençten Y . Carcinosarcomaof thebladder: a case report and review of the literature. Case Rep Urol 2013;2013:716704, 1, 3. 23956923 10.1155/2013/716704 PMC3728524

[9] Hoshi S , Sasaki M , Muto A , et al. Case of carcinosarcoma of urinary bladder obtained a pathologically complete response by neoadjuvant chemoradiotherapy. Int J Urol. 2007;14:79‐81. 17199866 10.1111/j.1442-2042.2006.01600.x

[10] Robinson SP , Farooq A , Laniado M , Motiwala H . The demographic features, clinical outcomes, prognosis and treatment options for patients with sarcomatoid carcinoma of theurinarybladder: a single centre experience. Int Braz J Urol. 2018;44:45‐52. 29064650 10.1590/S1677-5538.IBJU.2016.0347 PMC5815531

[11] Surveillance, Epidemiology, and End Results Program (SEER). National Cancer Institute. https://seer.cancer.gov/

[12] Wang J , Wang FW , Lagrange CA , et al. Clinical features of sarcomatoid carcinoma (carcinosarcoma) of the urinary bladder: analysis of 221 cases. Sarcoma 2010;2010:454792, 1, 7. 20706685 10.1155/2010/454792 PMC2913791

[13] Nazemi A , Daneshmand S . Adult genitourinary sarcoma: a population‐based analysis of clinical characteristics and survival. Urol Oncol. 2020;38:334‐343. 32094047 10.1016/j.urolonc.2019.12.004

[14] Malla M , Wang JF , Feng A , Wang J . Sarcomatoidcarcinomaof theurinarybladder. Clin Genitourin Cancer. 2016;14:366‐372. 27050715 10.1016/j.clgc.2016.03.004

[15] Mukhopadhyay S , Shrimpton AE , Nsouli IS , Abraham NZ Jr . Carcinosarcomaof theurinarybladderfollowingcyclophosphamide therapy: evidence for monoclonal origin and chromosome 9p allelic loss. Arch Pathol Lab Med. 2004;128:e8‐e11. 14692829 10.5858/2004-128-e8-COTUBF

[16] Dali KM , Kacem A , Ben Rhouma S , Chaker K Sellami A , Nouira Y . Carcino‐sarcoma of the urinary bladder with cartilaginous differentiation: about a case report. Urol Case Rep. 2019;28:101053. 31681537 10.1016/j.eucr.2019.101053 PMC6818139

[17] Hirano D , Yoshida T , Funakoshi D , Sakurai F , Ohno S , Kusumi Y . A case of early stage bladder carcinosarcoma in late recurrence of urothelial carcinoma after transurethral resection. Case Rep Urol. 2018;2018:1405108. 29568660 10.1155/2018/1405108 PMC5820658

[18] Lopez‐Beltran A , Pacelli A , Rothenberg HJ , et al. Carcinosarcoma and sarcomatoid carcinoma of the bladder: clinicopathological study of 41 cases. J Urol. 1998;159:1497‐1503. 9554341 10.1097/00005392-199805000-00023

[19] Wright JL , Black PC , Brown GA , et al. Differences in survival among patients with sarcomatoid carcinoma, carcinosarcoma and urothelial carcinoma of the bladder. J Urol. 2007;178:2302‐2306. 17936803 10.1016/j.juro.2007.08.038

